# Control of Silver Diffusion in Low-Temperature Co-Fired Diopside Glass-Ceramic Microwave Dielectrics

**DOI:** 10.3390/ma11010055

**Published:** 2017-12-29

**Authors:** Chen-Chia Chou, Chun-Yao Chang, Guang-Yu Chen, Wen-Jiao Liao, Kuei-Chih Feng, Chung-Ya Tsao

**Affiliations:** 1 Department of Mechanical Engineering, National Taiwan University of Science and Technology, No. 43, Keelung Road, Sec. 4, Taipei 10607, Taiwan; adimini08@gmail.com (C.-Y.C.); tp609803167@gmail.com (G.-Y.C.); 2 Department of Electrical Engineering, National Taiwan University of Science and Technology, No. 43, Keelung Road, Sec. 4, Taipei 10607, Taiwan; wjliao@mail.ntust.edu.tw; 3 Department of Mechanical Engineering, Ming Chi University of Technology, Taishan Dist., New Taipei City 24301, Taiwan; kwechin@mail.mcut.edu.tw; 4 Prosperity Dielectric Company Limited, Taoyuan 338, Taiwan; alberttsao@pdc.com.tw

**Keywords:** microwave dielectrics, silver diffusion, glass-ceramics, diopside, co-fired silver electrode, low temperature co-fired ceramics

## Abstract

Electrode material for low-temperature co-fired diopside glass-ceramic used for microwave dielectrics was investigated in the present work. Diffusion of silver from the electrode to diopside glass-ceramics degrades the performance of the microwave dielectrics. Two approaches were adopted to resolve the problem of silver diffusion. Firstly, silicon-oxide (SiO_2_) powder was employed and secondly crystalline phases were chosen to modify the sintering behavior and inhibit silver ions diffusion. Nanoscale amorphous SiO_2_ powder turns to the quartz phase uniformly in dielectric material during the sintering process, and prevents the silver from diffusion. The chosen crystalline phase mixing into the glass-ceramics enhances crystallinity of the material and inhibits silver diffusion as well. The result provides a method to decrease the diffusivity of silver ions by adding the appropriate amount of SiO_2_ and appropriate crystalline ceramics in diopside glass-ceramic dielectric materials. Finally, we used IEEE 802.11a 5.8 GHz as target specification to manufacture LTCC antenna and the results show that a good broadband antenna was made using CaMgSi_2_O_6_ with 4 wt % silicon oxide.

## 1. Introduction

The rapid growth of telecommunication and the satellite broadcasting industry have created a large demand for microwave ceramic components. To fulfill this demand, low temperature co-fired ceramic (LTCC) substrates integrated with passive devices have been used extensively for high-density packaging modules and high-performance wireless components in recent years. However, many material systems cannot meet the requirement of microwave dielectric components for the low temperature co-fired ceramics (LTCC) process due to their high sintering temperatures (>1300 °C). The low sintering temperature provided by LTCC technology is a key issue to be considered in microelectronic and microsystems and in microwave modules [1,2,3]. Therefore, LTCC materials with low permittivity have been developed in the form of glass-added ceramics (multiphase ceramics) and/or crystallizable glass [4,5,6].

High-conductivity metals, such as Ni, Cu, and Ag have been widely used as inner-electrode materials in LTCC components and modules to prepare multilayered LTCC devices. Since Ag can be sintered without atmosphere control, Ag metal and its alloys have been considered as highly effective inner-electrode materials in LTCC manufacturing [7]. Compatibility between the electrode and substrate, camber behavior of the sintered modules, and diffusion of silver ion during sintering are major issues for the metallization of LTCC components and modules. Camber behavior of multilayer structure co-firing with Ag paste has been intensive studied by Jean and co-workers [8,9]. Diffusion of Ag ions in high-glass content LTCC substrates was considered to influence reliability and performance of the LTCC modules due to increasing leakage current and decreasing insulation resistance.

Some silicate-based materials exhibit very high quality factor in the microwave range, and a CaO-MgO-SiO_2_ material system was prepared and investigated using the high temperature sintering method [10,11,12,13,14,15,16,17]. CaO-MgO-SiO_2_ forms a crystallized phase, named diopside and exhibits a low dielectric constant of less than 10 (we call it K10 here). Due to the open structure of the silicate network, phase transformation from amorphous to crystalline was attempted in our previous works [12,13]. Based upon those efforts, we further developed a crystallizable CaO-MgO-SiO_2_ material system for LTCC applications, which can be used in a low-temperature sintering route [11,12,13]. Although migration of silver ions in various electronic components and hybrid electronics were investigated earlier [18,19,20,21,22,23,24], the diffusion and reaction of silver ions in the glass-ceramic CaO-MgO-SiO_2_ substrate are not clear. In this study, diffusion kinetics of silver atoms/ions and microstructural characteristics in a glass-ceramic CaO-MgO-SiO_2_ microwave dielectric substrate were performed and here discussed.

## 2. Results and Discussion

**Figure 1:**
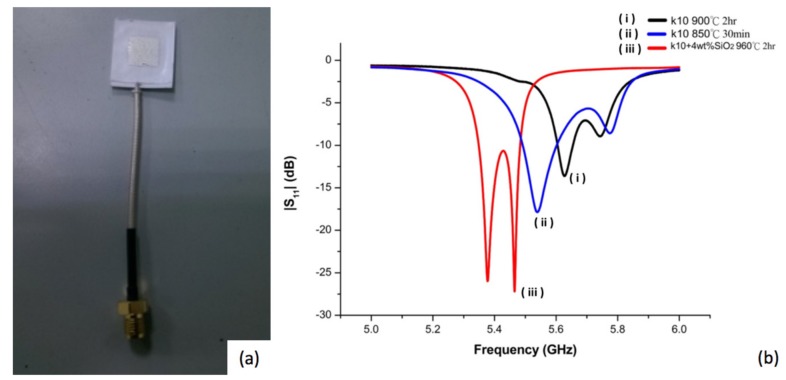
(**a**) Image showing fabricated microstrip antenna and (**b**) reflection coefficients for two different low temperature co-fired ceramic (LTCC) processing conditions of diopside glass-ceramics and for a modified diopside glass-ceramic material.

Fabrication and measurement of antenna impedance matching (reflection coefficient) was carried out. Figure 1a shows the image of a fabricated microstrip antenna and Figure 1b shows the impedance matching patterns for a designed antenna employing non-modified diopside glass ceramics using two different processing conditions. It was found that the reflection coefficient could be reduced (from −13.63 to −17.86) when the sintered parameters of the diopside glass-ceramics were switched from 900 °C for 2 h to 850 °C for 30 min, although the resonant frequency may move to low frequency (5.62 offset to 5.53), Figure 1b(i,ii). The results imply that the device properties and characteristics may be influenced by the material fabrication conditions. Since the scheme and design of the device are the same, the variation of the properties of the antenna can be attributed to material issues.

The main factor to influence the resonance frequency offset is the sintering shrinkage rate. The shorter sintering time and lower sintering temperature deliver smaller shrinkage of the device made of diopside glass-ceramics. Another material factor influencing device properties may be attributed to elemental diffusion. In the present case, silver was used as the electrode which may diffuse in the material and alter the properties of the antenna. In a previous experiment, it was reported that addition of SiO_2_ shows an effect of suppressing silver diffusion in the glass-added material system, and less diffusion of silver caused the reflection coefficient to decrease and thus better microwave signal absorbing components were obtained [7]. To verify this approach, we adopted nano-scale-SiO_2_ modified diopside powders (nano-silica dielectrics, NSK) to fabricate an antenna employing an LTCC process. The antenna design is the same as in previous work in Figure 1a,b, but the sintering temperature was raised to 960 °C for 2 h. The reflection coefficient can be reduced significantly (from −17.86 to −27.19) by adding 4 wt % of silicon oxide to the diopside glass-ceramic, Figure 1b(iii), which appears to be able to inhibit silver diffusion, although the resonant frequency of the antenna is shifted to 5.4 GHz. When the antenna element size of the green tape is the same, the electrode sizes are 10.1 mm for nano-sized SiO_2_ modified diopside antenna (NSK, 960 °C, 2 h), and 9.7 mm, 9.4 mm, for diopside (K10, 850 °C, 30 min) and (K10, 900 °C, 2 h). The bigger the electrode size after sintering, the more the electrode resonant frequency will move to lower frequency. In addition to this result, it can be deduced that with the lower sintering temperature or the shorter sintering time of the LTCC processing, the silver diffusion in the diopside can be reduced, and therefore deliver better devices.

**Figure 2:**
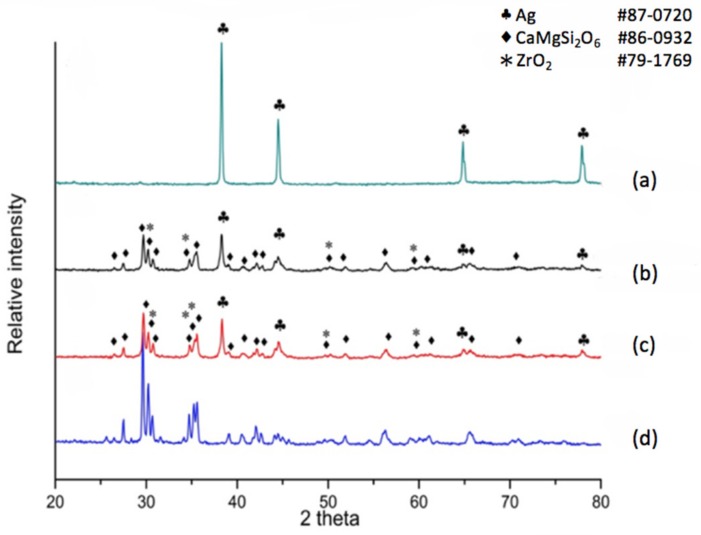
X-ray diffraction (XRD) patterns for different positions of a non-modified diopside CaO-MgO-2SiO_2_ system co-fired with a commercial silver electrode paste. (**a**) Electrode surface; (**b**) below surface 20 μm; (**c**) below surface 40 μm; (**d**) crystallized CaO-MgO-2SiO_2_ (diopside).

X-ray diffraction was also applied for the phase distribution and analysis of reactions due to silver diffusion. If the dielectric material is a non-modified diopside glass-ceramic material, a commercial silver electrode can be applied and co-fired. The phases at the electrode surface, electrode/dielectric boundary (20 μm below the electrode surface) and the plane 40 μm below the electrode surface were analyzed by X-ray diffraction. The results shown in Figure 2a indicate that the electrode surface exhibits a pure metallic silver structure, implying a good conductive material. The electrode/dielectric boundary, Figure 2b, consists of three phases: Diopside (CaMgSi_2_O_6_ # 86-0932), Zirconia (# 79-1769), Silver (# 87-0720). Thus we can see that the electrode boundary may contain a small part of silver due to diffusion. Similarly, three phases exist at the plane underneath the electrode surface at 40 μm, Figure 2c. The phases are Diopside (CaMgSi_2_O_6_ # 86-0932), Zirconia (# 79-1769), and Silver (# 87-0720). The diffraction pattern in Figure 2d shows the diffraction peaks of the diopside phase (K10) for comparison. We observe that a non-modified diopside glass-ceramic co-fired with the silver electrode shows serious silver diffusion into the dielectric material, even down to a depth of 40 μm.

**Figure 3:**
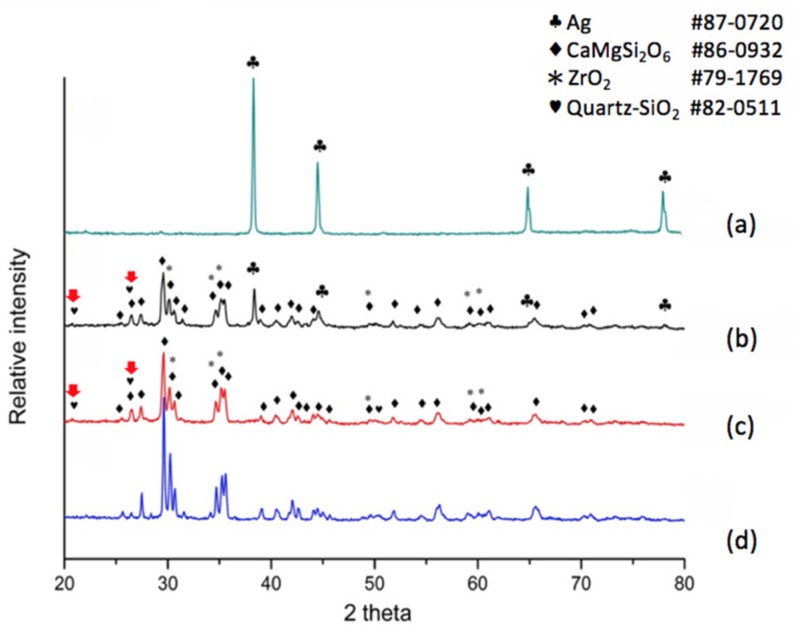
XRD patterns for different positions of a modified CaO-MgO-2SiO_2_ diopside system with nano-sized SiO_2_ addition co-fired with a commercial silver electrode. (**a**) electrode surface; (**b**) below surface 24 μm; (**c**) below surface 40 μm; (**d**) crystallized CaO-MgO-2SiO_2_ (diopside).

When the dielectric material was a modified diopside glass-ceramic material with 4 wt % nano-scaled SiO_2_ powder, we applied a commercial silver electrode and co-fired the material/device once more. The results shown in Figure 3a tell us that the electrode surface exhibits a face-centered cubic metallic silver structure again, showing a fully conductive function. The electrode/dielectric boundary, Figure 3b, consists of four phases: Diopside (CaMgSi_2_O_6_ # 86-0932), Zirconia (# 79-1769), Silver (# 87-0720), and Quartz (# 82-0511). Thus we can see that at the position a little lower than the electrode/dielectrics boundary a small amount of silver may be exhibited. However, only three phases were observed at the position of 40 μm beneath the electrode surface, Figure 3c, i.e., Diopside (CaMgSi_2_O_6_ # 86-0932), Zirconia (# 79-1769), and Quartz (# 82-0511). Comparing with the pure diopside phase, Figure 3d, it can be seen that when 4 wt % of silica was added to the diopside glass-ceramic, silver diffusion was retarded. In addition, X-ray diffraction analysis revealed that the originally added amorphous silicon oxide is transformed to the quartz phase due to heat treatment, and follow-up compositional analysis demonstrated further that the quartz formation suppressed silver diffusion.

**Figure 4:**
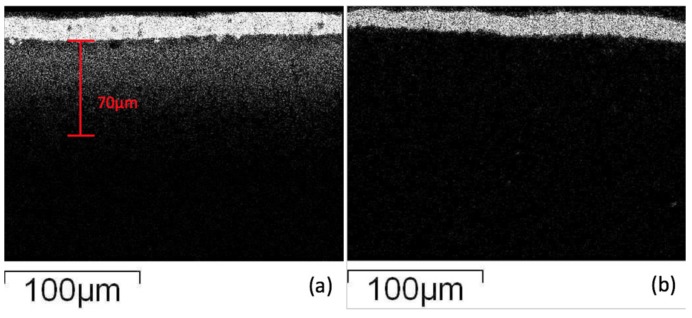
X-ray mapping of scanning electron microscopy (SEM)–energy dispersive X-ray spectroscopy (EDS) micrographs showing silver distribution for specimens of (**a**) CaO-MgO-2SiO_2_ stoichiometric composition; (**b**) modified CaO-MgO-2SiO_2_ system.

In order to observe the glass ceramic diffusion reaction, the results of the interfacial layer were investigated employing electron microscopy. Using elemental analysis (X-ray Mapping), the results (Figure 4) show that Ag appears at the electrode boundary and diffuses into the glass-ceramic at a distance of about 70 μm. The diffusion mechanism of silver may be as follows: the silver easily reacts with oxygen atoms in the glass phase at high temperatures, which creates a higher affinity of silver to the oxide matrix by a Ag–O bond, and the transportation effect of silver may be accompanied by element diffusion and crystallization in the glass-ceramic [24]. The diffusion of silver proceeds and may gather in block structures, and when the specimen cools down, silver may form a particulate precipitation in the glass-ceramic. On the other hand, the diopside glass-ceramic added with 4 wt % of SiO_2_ co-fired with silver electrode shows that the electrode/dielectric boundaries are much clearer, and no white spots appear in micrographs (high silver content) in the glass-ceramic substrate, implying that silver diffusion was suppressed in the materials. The results also indicate that it is more difficult for silver atoms to obtain oxygen atoms from an environment filled with nano-scale crystallized quartz particulates. The silver atoms were stopped at the electrode/dielectric boundary due to lack of affinity for the oxide environment.

**Figure 5:**
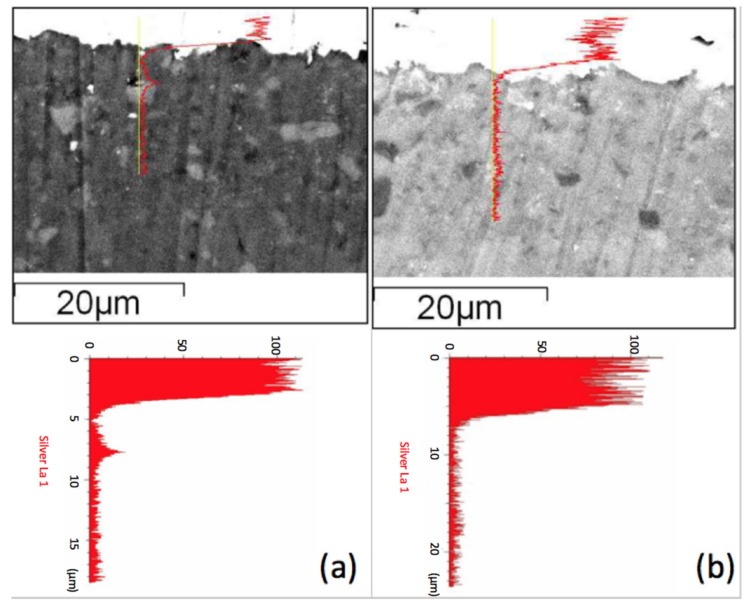
SEM micrograph and EDS curve showing silver distribution in modified CaO-MgO-2SiO_2_ system with (**a**) 8 wt % (Zn_0.6_Mg_0.4_)_2_SiO_4_ ceramics; (**b**) 4% SiO_2_.

**Figure 6:**
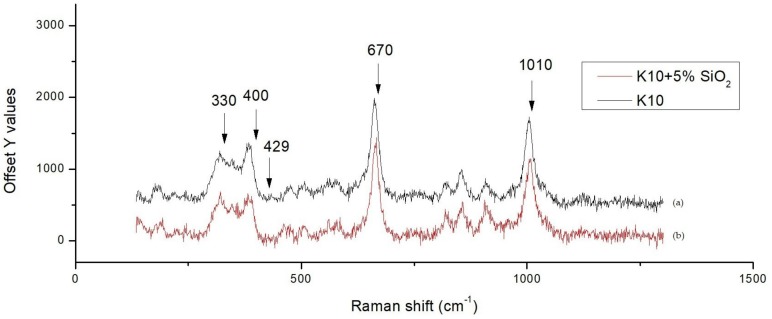
Raman spectra for (**a**) without SiO_2_ added diopside glass-ceramic; and (**b**) with SiO_2_ added diopside glass-ceramic.

Adding 8 wt % (Zn_0.6_Mg_0.4_)_2_SiO_4_ (ZMS) powder also shows the effect of reducing the silver diffusion, but not as good as with SiO_2_. Figure 5 shows the scanning electron microscopy (SEM) secondary electron images combined with line scanning traces on the position of detecting, where the line scanning profiles are shown and enlarged below the secondary electron images of two specimens. We observed a Ag peak at the interface of the ZMS article and the diopside glass-ceramics. This peak may be attributed to the amorphous phase in the dielectrics, which dissolves more of the Ag element. When taking the Raman spectrum, Figure 6, we observed a small peak of Ag–O bonding in material at 429 cm^−1^, implying that Ag may be at an oxidation state. Considering more carefully, the amorphous phase content in ZMS-modified diopside glass-ceramics appears to be higher than that in the SiO_2_-modified diopside glass-ceramics, because of the particle size and distribution.

When we prepare bulk specimens, samples with pure diopside phase or ZMS-modified diopside, the phase exhibits a white appearance, whereas on the other hand samples with SiO_2_-modified diopside glass-ceramics show a grey color, implying that some silica may be incorporated into the diopside lattice and introduce defects such as Mg or Ca deficient diopside or oxygen vacancies, which darken the ceramics. A possibility is that silver trapping occurred in the defects and then silver diffusion was inhibited. Four major peaks corresponding to the diopside phase shown in Figure 6 are at 330, 400, 670, and 1010 cm^−1^. If we overlap the Raman spectra, one finds that the 330 and 400 peak intensity changes and the 670 and 1010 intensity do not vary, indicating that silica addition into the diopside substrate actually modifies the vibration mode of the diopside lattice.

**Figure 7:**
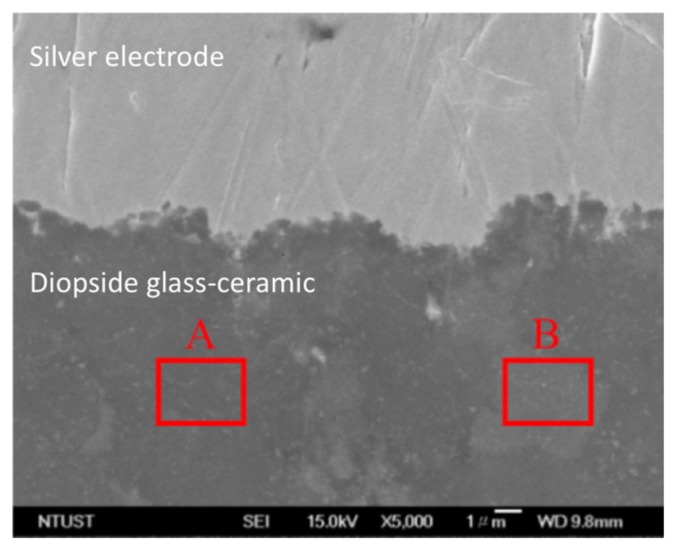
SEM micrograph and EDS data showing compositions corresponding to regions A and B.

A more quantitative composition analysis for 8 wt % ZMS ceramics modified CaO-MgO-2SiO_2_ system was carried out using EDS. The ZMS ceramics are crystalline, and when mixed into diopside glass ceramics, one may observe that ZMS crystallites are distributed in the glass-ceramics. Some regions show a large crystalline structure (such as region A in image) and some regions exhibit an amorphous glass behavior (region B) shown in Figure 7. Using area scanning, the composition of region A exhibiting crystalline characteristics, shows a low Ag concentration close to the background. On the other hand, region B is amorphous and the Ag composition is much higher shown in Table 1 and Table 2. The results are consistent with the line scanning result shown in Figure 5a, implying that the silver atomic distribution is more uniform in the NSK series of diopside-based glass-ceramic materials and the crystalline particulates form a Ag diffusion retardant.

## 3. Materials and Methods

LTCC substrates were prepared using diopside ceramic-glass powder. Diopside CaMgSi_2_O_6_ glass-ceramic was prepared through oxide mixing, melting, and heat treatments [11,12,13]. A mixture of CaCO_3_ (>99%, Komoshina, Tokyo, Japan), Mg(OH)_2_ (>99%, Ube, Tokyo, Japan) as well as SiO_2_ (>99%, Sibelco, Taichung, Taiwan) powders in a specific molar ratio of 1:1:2 and 3–11 wt % ZrO_2_ (>99.5%, Daiichi, Japan) were added as a nucleating agent for the mixtures by ball milling and vacuum drying at 60 °C. The resulting powders were melted at 1500 °C for 2 h, furnace cooled, pulverized, and ball milled, and then pressed under a loading of 1000 kgf to fabricate thin circular pellets (diameter of 15 mm) to test the fundamental properties. The controlled crystallization of glass-ceramic was carried out in a sintering temperature ranging from 800 to 950 °C for 4 h [13].

**Figure 8:**
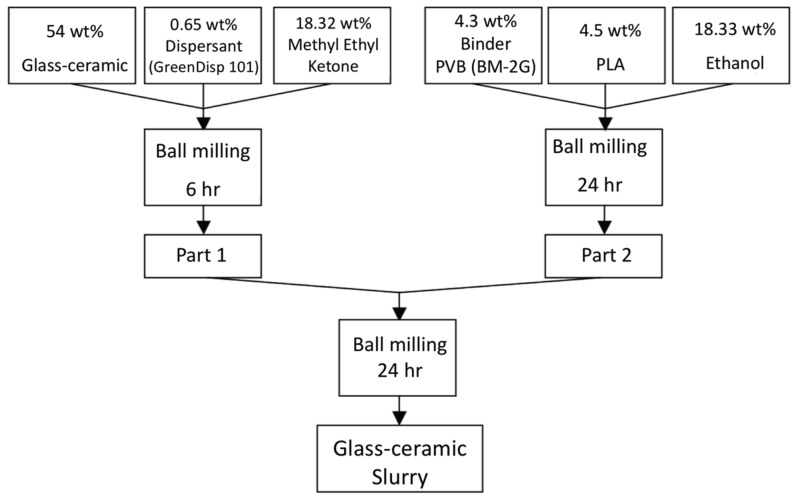
Preparation procedures for the LTCC pastes and content of the ingredients.

The procedures of powders mixed with organic binder and solvents is shown in Figure 8. Part A shows the glass-ceramic powders mixed with dispersant KD-1 and methyl ethyl ketone, and ball-milled for 6 h; Part B contains binder BM-2G, plasticizer PLA, and ethyl alcohol and ball-milled for 24 h, Part A and part B were mixed and ball-milled using zirconia balls for 24 h to prepare the slurry for the LTCC green tapes. Thick films with the desired thickness were fabricated using a tape casting method. Silver conducting paste was printed on the green tape and dried at 90 °C for 30 min. The printed green tapes were laminated at 90 °C under pressure of 200 kgf for 20 min in an isostatic pressing chamber. The diced samples were heated to 450 °C with a 5 °C/min heating rate for binder burnout before co-firing was conducted at temperatures between 800 and 950 °C for 30–120 min with a heating rate of 5 °C/min.

Cross-section samples were then polished using a diamond-containing plastic film to a surface roughness of 1 µm before etching with diluted 3% HF + HCl solution. Analysis of the microstructure was performed with scanning electron microscopy (SEM, JEOL 5600, Tokyo, Japan). Inter-diffusion between Ag and LTCC substrate of cross-sectioned samples was examined using a SEM equipped with an energy dispersive X-ray spectroscopy (EDS, Oxford 6587, Oxford, UK). X-ray mapping and line scanning were employed to reveal the distribution of elements. Crystalline phases of the co-fired dielectrics/devices were determined by X-ray diffractometry (XRD) using D2 phaser (Bruker, Karlsruhe, Germany). The phase analysis underneath the electrode was carried out using co-fired samples after precision polishing to remove a thin layer of material along the depth direction. Phases at the electrode surface, electrode/dielectric interface, glass ceramic dielectrics (40 μm below the electrode/dielectric interface) were evaluated by XRD.

To test the performance of the developed materials, we used IEEE 802.11a 5.8 GHz as the target specification to manufacture LTCC antenna, and measured the reflection coefficient of antenna using a network analyzer HP 8722A (Agilent, Santa Clara, CA, USA).

## 4. Conclusions

Silver-based electrode material for low-temperature co-fired diopside glass-ceramic used for microwave dielectrics was investigated in the present work. Diffusion of silver from the electrode to the diopside glass-ceramic degrades the performance of the microwave dielectrics. Two approaches were adopted to resolve the problem of silver diffusion. Results show that nanoscale amorphous SiO_2_ powder turns to the quartz phase uniformly in dielectric material during the sintering process and prevents the silver from diffusion. The chosen crystalline phase (Zn_0.6_Mg_0.4_)_2_SiO_4_ (ZMS) mixing into the glass-ceramics inhibits silver diffusion as well. The results demonstrate methods to decrease the diffusivity of silver ions by adding an appropriate amount of crystalline ceramic to the diopside glass-ceramic dielectric materials. Finally, we used IEEE 802.11a 5.8GHz as the target specification to manufacture LTCC antenna and the results show that a good broadband antenna can be made using CaMgSi_2_O_6_ with 4 wt % silicon oxide.

## Figures and Tables

**Table 1:** Composition of region A.

Element	Weight%	Atomic%
Mg K	13.24	19.93
Si K	34.50	44.95
Ca K	24.69	22.54
Zn K	10.66	5.97
Zr K	14.20	5.70
Ag K	2.72	0.92
Totals	100.00	

**Table 2:** Composition of region B.

Element	Weight%	Atomic%
Mg K	14.37	20.46
Si K	27.49	40.72
Ca K	24.07	21.68
Zn K	12.49	7.95
Zr K	10.94	4.99
Ag K	10.89	4.20
Totals	100.00	
